# Musculoskeletal Soft-Tissue Sarcoma: Quality Assessment of Initial MRI Reports Shows Frequent Deviation from ESSR Guidelines

**DOI:** 10.3390/diagnostics11040695

**Published:** 2021-04-14

**Authors:** Sebastian Weiss, Alexander Korthaus, Nora Baumann, Jin Yamamura, Alexander S. Spiro, Andreas M. Lübke, Karl-Heinz Frosch, Carsten Schlickewei, Matthias Priemel

**Affiliations:** 1 Department of Trauma and Orthopaedic Surgery, University Medical Center Hamburg-Eppendorf, 20246 Hamburg, Germany; a.korthaus@uke.de (A.K.); nora.baumann@stud.uke.uni-hamburg.de (N.B.); a.spiro@uke.de (A.S.S.); k.frosch@uke.de (K.-H.F.); c.schlickewei@uke.de (C.S.); priemel@uke.de (M.P.); 2 Department of Diagnostic and Interventional Radiology and Nuclear Medicine, University Medical Center Hamburg-Eppendorf, 20246 Hamburg, Germany; j.yamamura@uke.de; 3 Department of Pediatric Orthopedics, Children’s Hospital Hamburg Altona, 22763 Hamburg, Germany; 4 Institute of Pathology, University Medical Center Hamburg-Eppendorf, 20246 Hamburg, Germany; luebke@uke.de; 5 Department of Trauma Surgery, Orthopaedics and Sports Traumatology, BG Klinikum Hamburg, 21033 Hamburg, Germany

**Keywords:** soft-tissue sarcoma, MRI, report, radiology, MSK, sarcoma center, ESSR, unplanned excision, biopsy

## Abstract

Soft-tissue sarcomas (STS) are a rare subtype of soft-tissue mass and are frequently misinterpreted as benign lesions. Magnetic resonance imaging (MRI) is the primary recommended type of diagnostics. To assess the quality of primary radiology reports, we investigated whether recommended MRI report elements were included in compliance with European Society of Musculoskeletal Radiology (ESSR) guidelines. A total of 1107 patients were evaluated retrospectively, and 126 radiological reports on patients with malignant STS were assessed for ESSR quality criteria. One or more required sequences or planes were missing in 67% of the reports. In all 126 cases, the report recognized the mass as anomalous (100%). Sixty-eight percent of the reports mentioned signs of malignancy. The majority of reports (*n* = 109, 87%) articulated a suspected diagnosis, 32 of which showed a mismatch with the final diagnosis (25%). Thirty-two percent of the reports had a misinterpretation of the masses as benign. Benign misinterpretations were more common in masses smaller than 5 cm (65% vs. 27%). Thirty percent of the reports suggested tissue biopsy and 6% recommended referral to a sarcoma center. MRI reports showed frequent deviations from ESSR guidelines, and protocol guidelines were not routinely met. Deviations from standard protocol and reporting guidelines could put patients at risk for inadequate therapy.

## 1. Introduction

Soft tissue sarcomas (STS) are a rare subtype of soft-tissue mass, representing less than 1% of all malignancies [1]. The incidence in Europe is estimated at 3.6–4.7 cases per 100,000 people every year [2,3,4]. Due to their rareness, soft-tissue sarcomas are frequently mistaken for benign soft-tissue lesions, which are 300 times more common [5]. Sensitivity of detecting soft-tissue sarcoma out of all soft-tissue masses through MRI imaging has been described at only 80% in prospective studies [6]. In many cases, misdiagnosis leads to unplanned excisions and, therefore, to inadequate therapy.

To optimize the outcome for patients with soft-tissue sarcomas, treatment at specialized sarcoma centers is strongly recommended [7,8,9,10]. Otherwise, adherence to the principles of good clinical practice in the treatment of soft-tissue sarcoma might be compromised, leading to a significantly increased rate of local recurrence and reduced overall survival [11,12].

In cases of suspicious soft-tissue masses, magnetic resonance imaging (MRI) is the primary recommended type of examination. Therefore, subsequent radiologic reports play a crucial role in the detection of soft-tissue sarcomas and their following path of treatment [1]. In any case of an indeterminate lesion, surgical oncology consultation and consequent planned tissue biopsy comprise the next mandatory diagnostic step [13,14]. Unplanned excisions of STS without prior biopsies bear the risk of incomplete resection and have shown a significantly reduced five-year recurrence-free survival compared to planned excisions (63.7% vs. 89.7%) [15].

According to the European Society for Musculoskeletal Radiology (ESSR) guidelines, radiological reports on soft tumor masses should describe the exact anatomical location and use fluid-sensitive fat-saturated and T1-weighted sequences in at least two planes with the additional use of a contrast agent [16]. Furthermore, reports should mention signs of malignancy and recommend obtaining a biopsy for histopathological evaluation and referral to a specialized sarcoma center.

The aim of this study was to evaluate whether the aforementioned requirements stated in the current ESSR guidelines were included in the primary radiologic description of findings in patients that were subsequently treated for soft-tissue sarcoma in our specialized center.

## 2. Materials and Methods

This study was approved by the ethics committee of the regional medical association (WF-095/20).

A total of 1107 patients presenting to the traumatology and orthopedic surgery department of a tertiary sarcoma center for biopsy or excision surgery from 2012 to 2018 were evaluated retrospectively. Patients presenting to another surgical department of the sarcoma center were not included.

**Figure 1:**
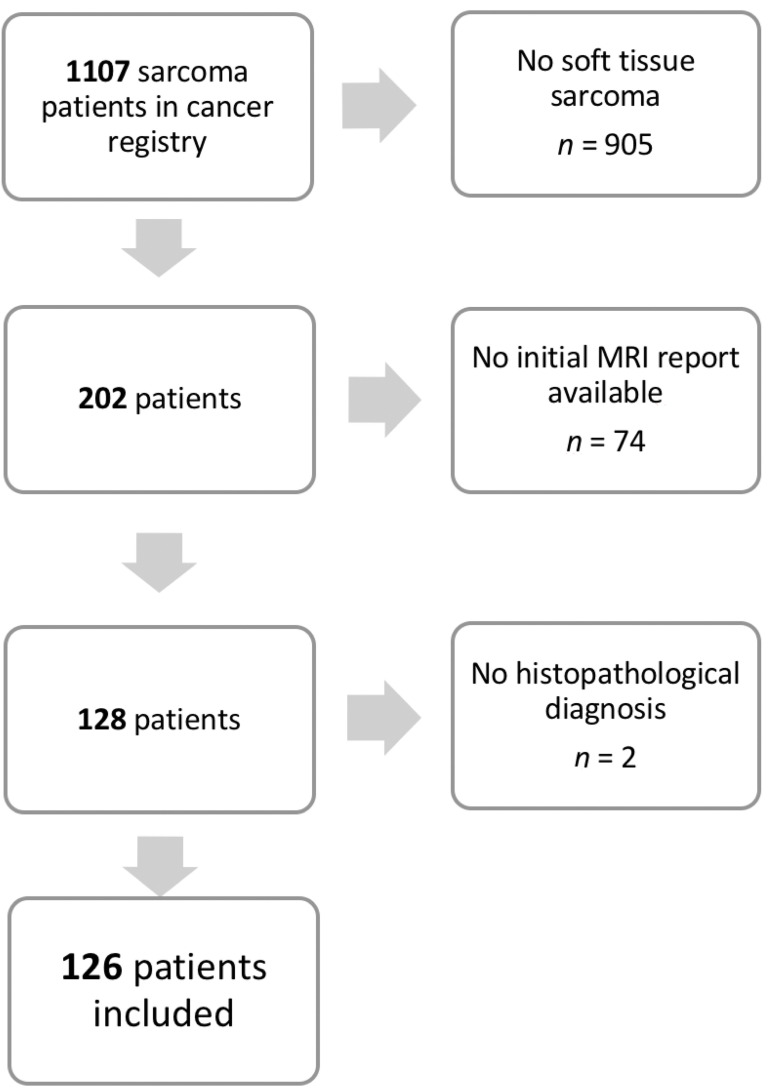
Flow chart of our study cohort. After removal of duplicates, 1107 patients with soft tissue sarcomas were listed in our cancer registry for biopsy or excision surgery between 2012–2018.

As a first step, patients with a definitive histopathological diagnosis of malignant soft-tissue sarcoma of the extremities were identified, excluding any subtypes of benign tumors, tumors with inconclusive histopathological results, and osseous sarcoma (*n* = 905). In some patient files, the initial MRI reports (*n* = 74) or final histopathological results (*n* = 2) could not be retrieved. Ultimately, 126 patients with complete initial MRI reports and final histopathological results were included in this study (Figure 1).

Underlying MRI images were not available on a regular basis and were not reevaluated.

The radiologic reports were analyzed for the following characteristics:

1. Was the soft-tissue mass detected and described as anomalous?
2. Which sequences (T1-weighted, T2-weighted, fluid-sensitive/fat-saturated) were performed, which planes (coronal, sagittal, axial) were included, and was a contrast agent used?
3. Was a conclusion drawn regarding the specific suspected diagnosis?
4. Did the report mention a suspected malignancy or signs thereof? In cases of suspected “sarcoma” (see question 3), this question was answered with “yes”.
5. Did the report include a recommendation for biopsy and histopathological analysis of the described tissue?
6. Was a referral to a specialized sarcoma center recommended?

Authors of MRI reports were listed, and their reports were counted to evaluate the number of contributed radiological reports from each radiologist or practice. Furthermore, the initial radiologically suspected diagnosis was compared to the final histopathological result to detect apparent mismatches. Mismatches were defined as differences in tumor dignity or in cases of a different suspected malignancy than the ultimate histopathological diagnosis. Vague suspected diagnoses (e.g., “sarcoma”) that were later specified into a subtype (e.g., “myxofibrosarcoma”) by histological evaluation were not considered a mismatch.

Statistical analysis was performed as a percentage-based evaluation, showing the proportions of cases in which the above-mentioned criteria were met.

## 3. Results

A total of 126 patients with a histopathologically confirmed soft-tissue sarcoma were included in our study. The study cohort consisted of 62 (49%) male and 64 (51%) female patients with a mean age of 63.1 (±17.5) years.

The most common subtypes of soft-tissue sarcomas were atypical lipomatous tumors/liposarcoma grade 1 (*n* = 24; 19%), followed by myxofibrosarcomas (*n* = 16; 13%), and myxoid liposarcomas (Table 1).

Radiological reports were obtained from 61 different radiological departments or medical practices. The largest share was from the in-house radiological department (*n* = 18); no other radiologist was responsible for more than six reports. Most medical practices contributed one or two reports to our patient cohort.

**Figure 2:**
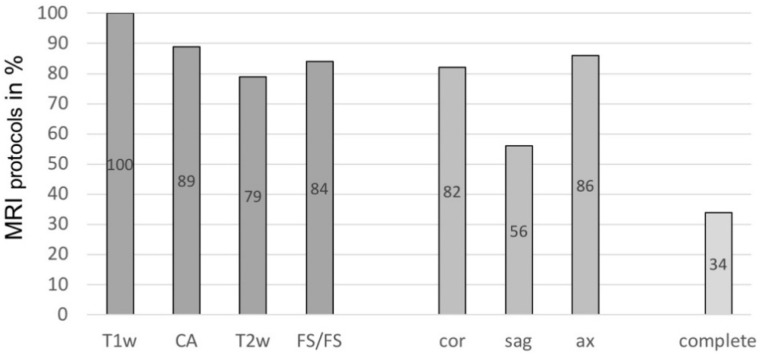
Percentage of European Society for Musculoskeletal Radiology (ESSR) recommended sequences and planes included in magnetic resonance imaging (MRI) protocols. T1w = T1-weighted; CA = use of contrast agent; T2w = T2-weighted; FS/FS = fluid-sensitive/fat-saturated sequence; cor = coronal plane; sag = sagittal plane; ax = axial plane; complete = all sequences/planes included.

In our study group, there were significant inconsistencies regarding the conducted MRI sequences (Figure 2). All reports (100%) performed a T1-weighted native sequence, and additional contrast agent was used in 89% of the MRIs. A fluid-sensitive, fat-saturated sequence was conducted in 84%, and T2-weighted sequences were carried out in 79%. A coronal plane was available in 82% of cases, axial plane in 86%, and sagittal in 56%, respectively.

Overall, only 34% of reports showed a complete MRI protocol that included all of the above-mentioned sequences and planes, whereas 66% of reports lacked one or more of these features.

**Figure 3:**
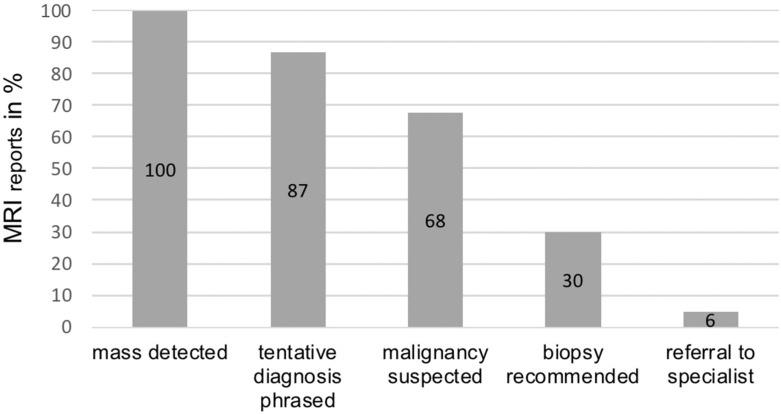
Percentage of MRI reports fulfilling ESSR guideline criteria.

Analysis of the initial MRI reports showed the following results (Figure 3):

The analysis of the initial MRI reports (Figure 3) demonstrated that in all 126 patients, the primary radiological report recognized and described the suspected mass as anomalous (100%). Eighty-six of the 126 radiologic reports raised concerns that the described mass showed signs of malignancy (68%) with some variation in certainty, ranging from mentioning indications of malignancy to a specific suspected sarcoma subtype.

Regardless of their categorization of masses as benign or malignant, the vast majority of reports (*n* = 109, 87%) phrased a specific suspected diagnosis. Thirty-two of these suspected diagnoses showed a gross mismatch with the final histopathological results, not counting reports in which the suspected diagnosis was broadly phrased and later specified through final histology (i.e., suspected “sarcoma”, with final diagnosis of “myxofibrosarcoma”).

**Figure 4:**
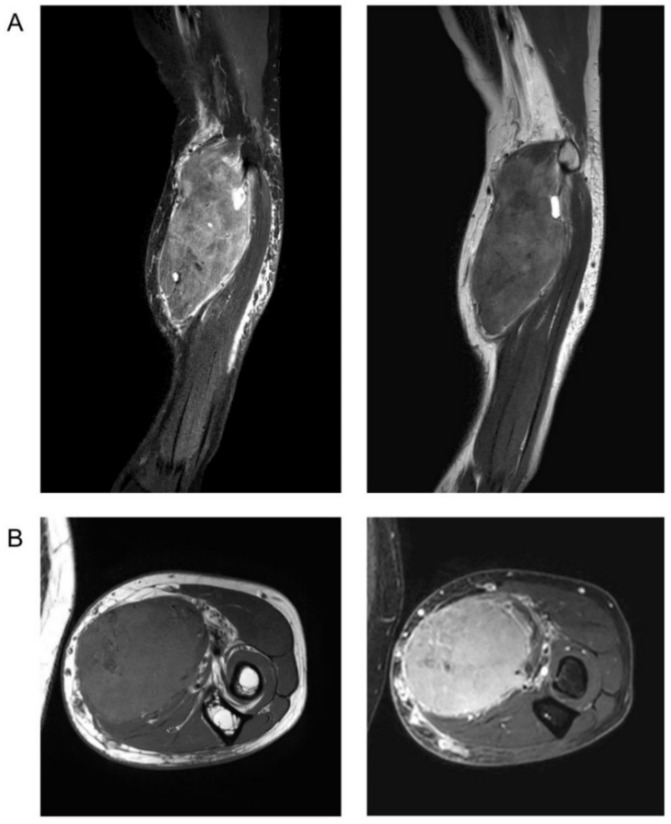
MRI of a patient with a suspected benign neurofibroma according to the initial MRI report. Histopathology after biopsy showed high-grade spindle cell sarcoma (NOS). (**A**). Sagittal T2-weighted sequence with fat saturation (left) and without fat saturation (right). (**B**). Axial T1-weighted sequence before (left) and after application of contrast agent with fat saturation (right).

**Figure 5:**
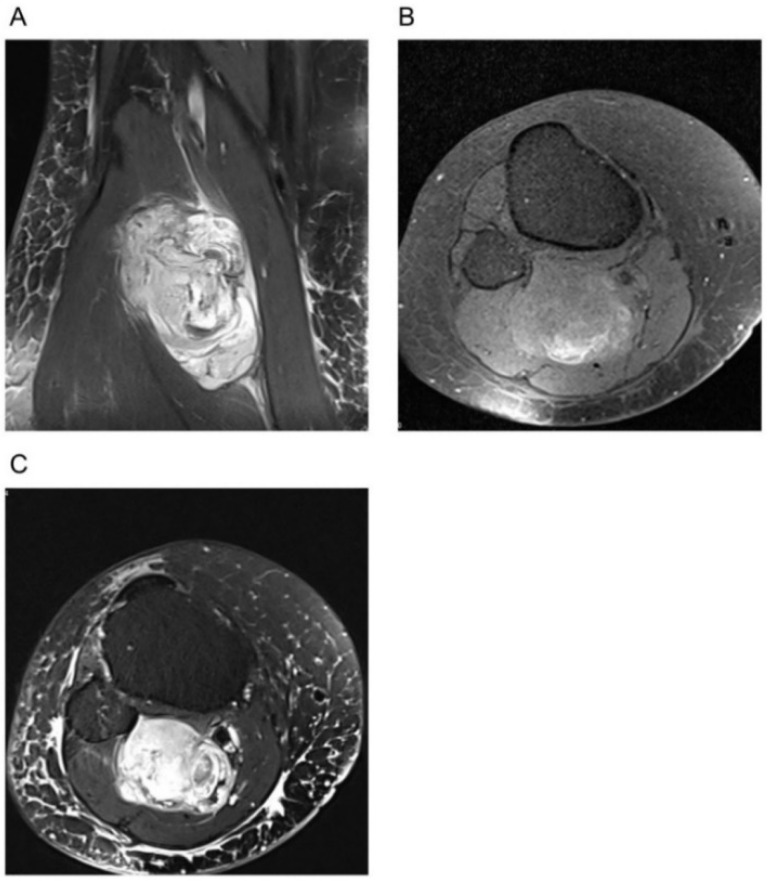
Initial MRI describing a suspected schwannoma; histology after resection confirmed malignant peripheral nerve sheath tumor (MPNST). (**A**) Coronal proton density (PD) sequence. (**B**) Axial fat-saturated T1 sequence without contrast agent. (**C**) Axial T2 sequence.

Five cases were correctly suspected to be malignant, but the suspected type of malignancy was different from the final result (3.9%). Forty reports contained a gross misinterpretation of masses as benign (32%), although the masses were malignant soft-tissue sarcomas in pathological findings (Figure 4 and Figure 5).

**Figure 6:**
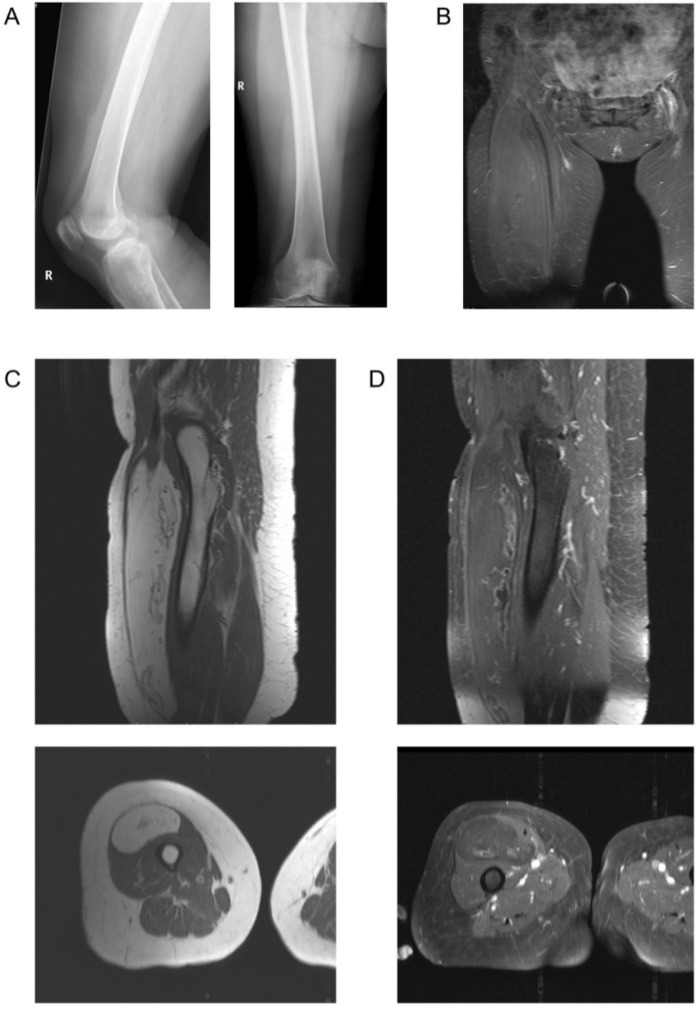
Images of a patient with low-grade liposarcoma (atypical lipomatous tumor) and an initial report describing lipoma. (**A**) X-Ray in lateral and a.p. view. (**B**) Coronal TIRM (STIR) sequence. (**C**) T1-weighted image precontrast in sagittal and axial plane. (**D**) T1-weighted image postcontrast with fat saturation.

Mass size appeared to have an influence on the interpretation of masses as benign or malignant. Large masses with a diameter of more than 5 cm in any plane were subject to a smaller number of false interpretations as benign (29 of 109 reports, 27%) than smaller masses (11 of 17 cases, 65%). The most common mismatch was a suspected lipoma (*n* = 10) when in fact histology after biopsy or excision showed liposarcoma (*n* = 9, 7 of which were well-differentiated liposarcoma grade I) or spindle-shaped sarcoma (*n* = 1) (Figure 6).

Thirty-eight reports specifically recommended a biopsy of the described mass for further analysis (30%). Only 7 reports recommended a referral to a specialized sarcoma or oncology center for further diagnostics and/or treatment (6%).

## 4. Discussion

Our study showed that various quality criteria as outlined in current guidelines are frequently not included in initial MRI reports of soft-tissue sarcomas.

Due to their rare incidence, many physicians, including primary-care physicians, orthopedic, plastic- or general surgeons, and radiologists that practice outside specialized sarcoma centers are only infrequently confronted with soft-tissue sarcomas [2]. Clinically, patients often present with a painless mass that has shown growth over an uncertain amount of time. Because sarcomas tend to have a deeper localization than benign lesions, their discovery is often delayed [17]. Pain as an additional symptom is usually related to infiltration of nerval structures or massive size [18].

Other, more common causes of soft-tissue masses include trauma, infection, benign lesions (especially lipoma), or metastases. Accompanying symptoms such as fever, night sweat, or weight loss are only rarely observed [19]. These unspecific clinical characteristics make an early suspected diagnosis of sarcoma difficult. Instead, the more common clinical assumption is that the observed mass is benign. For further diagnostics, patients with an unclear soft-tissue mass are regularly referred for radiological imaging.

MRI facilitates not only detection, but also reliable differentiation between low-grade and high-grade soft-tissue sarcoma [20,21]. However, with magnetic resonance imaging being the primary recommended imaging method for further diagnostics of suspicious soft-tissue masses, executing radiologists face the risk of neglecting sarcomas in their considerations due to their rareness [16,22,23]. In our study, which only included cases with histopathologically confirmed soft-tissue sarcoma, all radiologic reports described the observed mass, yet only 68% suspected it to be malignant or at least stated indications thereof.

Problems arise when soft-tissue masses are prematurely suspected to be benign. In our study, 40 reports (32%) did not articulate a suspected malignancy and 27 reports (21%) even described a specific benign tumor, whereas the final histological results proved malignancy. This inconsistency can have drastic consequences, as unplanned excisions of soft-tissue sarcoma often lead to incomplete resection and therefore hinder adequate disease control [24]. The most common example in our study is of cases where the radiological report suspected lipoma (10 cases) while the final histological result showed the presence of spindle cell sarcoma (1 case) or liposarcoma (9 cases) (Figure 6). Seven of these cases were in fact well-differentiated liposarcoma/liposarcoma grade I. Due to overlapping MRI characteristics, differentiation between lipoma and (well-differentiated) liposarcoma can be difficult [25,26,27]. In contrast to other soft-tissue sarcoma subtypes, a misinterpretation of low-grade liposarcoma as lipoma only carries minor clinical relevance, as treatment of lipoma and grade I liposarcoma is similar, and they are usually marginally resected [28].

Surprisingly, “benign” misinterpretation is not only an occurrence with small-size tumors, as there were several cases of large masses (>5 × 5 cm) in which the primary assessment was “benign” but later corrected to “malignant sarcoma” through histopathological analysis.

In the authors’ opinion, radiological reports should refrain from articulating a specific suspected diagnosis in cases of indeterminate soft-tissue masses. Instead, only signs of malignancy should be phrased, ideally in combination with the recommendation to obtain a tissue biopsy for further histopathological analysis. This process would be in line with the ESSR guidelines [16] and could reduce the risk of a mistaken preliminary suspicion and its consequences [29]. In our study, only 38 reports (30%) promoted a histopathological differentiation.

Furthermore, biopsies should be carried out in specialized sarcoma centers [30]. This enables initial biopsy and final resection in cases of malignancies to be performed by the same experienced surgeon [31]. Specialized sarcoma centers and their surgeons are experienced in the field of operative sarcoma therapy, and therefore, the risk of inadequate resection is reduced [7,8,32]. Additionally, adjuvant radiation and/or chemotherapy can be carried out in the same institution, which prevents loss of information and enables close interdisciplinary collaboration. In our study group, only 7 radiological reports (6%) recommended patient admission to a specialized sarcoma center. More frequent referrals could prevent delayed diagnosis and treatment.

The large number of different radiologists who contributed to our patient cohort’s radiological reports suggests that the issue of non-adherence to ESSR guidelines is widespread and not due to the lack of awareness of a few radiologists.

The broad variability in the conducted MRI sequences impedes the establishment of a common ground for diagnostics, interpretation, and treatment in cases of soft-tissue masses. Omission of certain sequences can lead to overlooked signs of malignancy. Additionally, incomplete plane reconstructions cause difficulty in the planning of surgical biopsy and especially ultimate excision, increasing the risk of subtotal excision or injury to surrounding structures.

The relationship between patient outcomes with respect to survival, local recurrence, or complications after surgery and the effect of initial MRI protocols and report adherence versus non-adherence to ESSR guidelines remains unclear. An effect on patient outcome would suggest that magnetic resonance imaging should be repeated in accordance with ESSR guidelines prior to any further treatment.

Only radiological reports based on conducted MRIs were evaluated in this study, because external MRI images themselves were not available in most cases due to lack of long-term file storage of external MRI images in our sarcoma center’s database. Image reevaluation by the authors and musculoskeletal radiology specialists would certainly provide further insights. However, for many primary care physicians or orthopedists, MRI reports, rather than the images themselves, are often the determining set of information used in deciding on further patient treatment or referral. This study reflects the data of the department of trauma and orthopedic surgery at a single sarcoma center. While the authors’ sarcoma center has a large catchment area in its region, evaluations of MRI reporting criteria might show different results in other countries or healthcare systems outside of the European continent with different existing guidelines. Furthermore, it is unknown whether some radiologists might have had other images (ultrasound, plain radiographs, computed tomography) to compare the conducted MRI to. The presence of such additional information would most likely have affected their diagnostic accuracy.

## 5. Conclusions

The present study identified substantial deviations of MRI protocols and reports from ESSR guidelines that may result in inadequate treatment of patients at risk. MRI reports in cases of soft-tissue masses should be based on a standardized protocol and written according to the guidelines, as such practices will be essential to ensuring high-quality reports and optimized care for patients in the future.

## Figures and Tables

**Table 1:** Numbers of specific subtypes of soft-tissue sarcomas, according to final histopathological reports.

Subtype of Soft-Tissue Sarcoma	Number of Cases
Atypical lipomatous tumor/liposarcoma grade I	24
Myxofibrosarcoma	16
Myxoid liposarcoma	15
Dedifferentiated liposarcoma	9
Synovial sarcoma	8
Fibromyxoid sarcoma	6
Pleomorphic undifferentiated sarcoma	7
Leiomyosarcoma	5
Malignant peripheral nerve sheath tumor(MPNST)	5
Pleomorphic spindle cell sarcoma (PSCS)	4
Undifferentiated pleophormic sarcoma	4
Epithelioid sarcoma	4
Undifferentiated sarcoma NOS	3
Angiosarcoma	2
Desmoplastic small-round-cell tumor	2
Spindle cell sarcoma NOS	2
Alveolar rhabdomyosarcoma	1
Extraskelettal osteosarcoma	1
Glomus tumor	1
High-grade myxoid liposarcoma	1
Clear-cell sarcoma	1
Myofibroblastic sarcoma	1
Pleomorphic liposarcoma	1
Pleomorphic rhabdomyosarcoma	1
Peripheral primitive neuroectodermal tumor (pPNET)	1
Sclerosing epithelioid fibrosarcom	1

## References

[1] Manaster B.J. (2013). Soft-tissue masses: Optimal imaging protocol and reporting. AJR Am. J. Roentgenol..

[2] Gatta G., van der Zwan J.M., Casali P.G., Siesling S., Dei Tos A.P., Kunkler I., Otter R., Licitra L., Mallone S., Tavilla A. (2011). Rare cancers are not so rare: The rare cancer burden in Europe. Eur. J. Cancer.

[3] Casali P.G., Abecassis N., Aro H.T., Bauer S., Biagini R., Bielack S., Bonvalot S., Boukovinas I., Bovee J., Brodowicz T. (2018). Soft tissue and visceral sarcomas: ESMO-EURACAN Clinical Practice Guidelines for diagnosis, treatment and follow-up. Ann. Oncol..

[4] Ducimetiere F., Lurkin A., Ranchere-Vince D., Decouvelaere A.V., Peoc’h M., Istier L., Chalabreysse P., Muller C., Alberti L., Bringuier P.P. (2011). Incidence of sarcoma histotypes and molecular subtypes in a prospective epidemiological study with central pathology review and molecular testing. PLoS ONE.

[5] Giuliano A.E., Eilber F.R. (1985). The rationale for planned reoperation after unplanned total excision of soft-tissue sarcomas. J. Clin. Oncol..

[6] Gielen J.L., De Schepper A.M., Vanhoenacker F., Parizel P.M., Wang X.L., Sciot R., Weyler J. (2004). Accuracy of MRI in characterization of soft tissue tumors and tumor-like lesions. A prospective study in 548 patients. Eur. Radiol..

[7] Pretell-Mazzini J., Barton M.D., Conway S.A., Temple H.T. (2015). Unplanned excision of soft-tissue sarcomas: Current concepts for management and prognosis. J. Bone Jt. Surg. Am..

[8] Gutierrez J.C., Perez E.A., Moffat F.L., Livingstone A.S., Franceschi D., Koniaris L.G. (2007). Should soft tissue sarcomas be treated at high-volume centers? An analysis of 4205 patients. Ann. Surg..

[9] Paszat L., O’Sullivan B., Bell R., Bramwell V., Groome P., Mackillop W., Bartfay E., Holowaty E. (2002). Processes and outcomes of care for soft tissue sarcoma of the extremities. Sarcoma.

[10] Clasby R., Tilling K., Smith M.A., Fletcher C.D. (1997). Variable management of soft tissue sarcoma: Regional audit with implications for specialist care. Br. J. Surg..

[11] Jakob J., Henzler T., Kasper B., Marx A., Hohenberger P. (2014). Interdisciplinary treatment of soft tissue sarcoma of the extremities. Chirurg.

[12] Blay J.Y., Honore C., Stoeckle E., Meeus P., Jafari M., Gouin F., Anract P., Ferron G., Rochwerger A., Ropars M. (2019). Surgery in reference centers improves survival of sarcoma patients: A nationwide study. Ann. Oncol..

[13] Gilbert N.F., Cannon C.P., Lin P.P., Lewis V.O. (2009). Soft-tissue sarcoma. J. Am. Acad. Orthop. Surg..

[14] Choi J.H., Ro J.Y. (2020). Retroperitoneal Sarcomas: An Update on the Diagnostic Pathology Approach. Diagnostics.

[15] Potter B.K., Adams S.C., Pitcher J.D., Temple H.T. (2008). Local recurrence of disease after unplanned excisions of high-grade soft tissue sarcomas. Clin. Orthop. Relat. Res..

[16] Noebauer-Huhmann I.M., Weber M.A., Lalam R.K., Trattnig S., Bohndorf K., Vanhoenacker F., Tagliafico A., van Rijswijk C., Vilanova J.C., Afonso P.D. (2015). Soft Tissue Tumors in Adults: ESSR-Approved Guidelines for Diagnostic Imaging. Semin. Musculoskelet. Radiol..

[17] Mayerson J.L., Scharschmidt T.J., Lewis V.O., Morris C.D. (2015). Diagnosis and Management of Soft-tissue Masses. Instr. Course Lect..

[18] George A., Grimer R. (2012). Early symptoms of bone and soft tissue sarcomas: Could they be diagnosed earlier?. Ann. R Coll. Surg. Engl..

[19] Damron T.A., Beauchamp C.P., Rougraff B.T., Ward W.G. (2004). Soft-tissue lumps and bumps. Instr. Course Lect..

[20] Sedaghat S., Ravesh M.S., Sedaghat M., Both M., Jansen O. (2021). Configuration of soft-tissue sarcoma on MRI correlates with grade of malignancy. Radiol. Oncol..

[21] Yan R., Hao D., Li J., Liu J., Hou F., Chen H., Duan L., Huang C., Wang H., Yu T. (2021). Magnetic Resonance Imaging-Based Radiomics Nomogram for Prediction of the Histopathological Grade of Soft Tissue Sarcomas: A Two-Center Study. J. Magn. Reson. Imaging.

[22] Wu J.S., Hochman M.G. (2009). Soft-tissue tumors and tumorlike lesions: A systematic imaging approach. Radiology.

[23] Ulaner G., Hwang S., Landa J., Lefkowitz R.A., Panicek D.M. (2013). Musculoskeletal tumours and tumour-like conditions: Common and avoidable pitfalls at imaging in patients with known or suspected cancer: Part B: Malignant mimics of benign tumours. Int. Orthop..

[24] Gingrich A.A., Elias A., Michael Lee C.Y., Nakache Y.N., Li C.S., Shah D.R., Boutin R.D., Canter R.J. (2017). Predictors of residual disease after unplanned excision of soft tissue sarcomas. J. Surg. Res..

[25] Brisson M., Kashima T., Delaney D., Tirabosco R., Clarke A., Cro S., Flanagan A.M., O’Donnell P. (2013). MRI characteristics of lipoma and atypical lipomatous tumor/well-differentiated liposarcoma: Retrospective comparison with histology and MDM2 gene amplification. Skeletal. Radiol..

[26] Sato D., Suga H., Takushima A. (2018). Liposarcoma Preoperatively Diagnosed as Lipoma: 10-Year Experience at a Single Institution. Dermatol. Surg..

[27] Thornhill R.E., Golfam M., Sheikh A., Cron G.O., White E.A., Werier J., Schweitzer M.E., Di Primio G. (2014). Differentiation of lipoma from liposarcoma on MRI using texture and shape analysis. Acad. Radiol..

[28] Choi K.Y., Jost E., Mack L., Bouchard-Fortier A. (2020). Surgical management of truncal and extremities atypical lipomatous tumors/well-differentiated liposarcoma: A systematic review of the literature. Am. J. Surg..

[29] De La Hoz Polo M., Dick E., Bhumbra R., Pollock R., Sandhu R., Saifuddin A. (2017). Surgical considerations when reporting MRI studies of soft tissue sarcoma of the limbs. Skeletal. Radiol..

[30] Dangoor A., Seddon B., Gerrand C., Grimer R., Whelan J., Judson I. (2016). UK guidelines for the management of soft tissue sarcomas. Clin. Sarcoma Res..

[31] Traina F., Errani C., Toscano A., Pungetti C., Fabbri D., Mazzotti A., Donati D., Faldini C. (2015). Current concepts in the biopsy of musculoskeletal tumors. J. Bone Jt. Surg. Am..

[32] Honore C., Meeus P., Stoeckle E., Bonvalot S. (2015). Soft tissue sarcoma in France in 2015: Epidemiology, classification and organization of clinical care. J. Visc. Surg..

